# A project designed to examine, for the first time, the health records of adult prisoners in Northern Ireland and their linkage to other available health data: the test case of prisoner post-release mortality risk.

**DOI:** 10.23889/ijpds.v7i3.2057

**Published:** 2022-08-25

**Authors:** Janine Cooper, Dermot O'Reilly, Richard Kirk, Trish Kelly, Rachel Gibbs, Michael Donnelly

**Affiliations:** 1 Queen's University Belfast; 2 South Eastern Health and Social Care Trust

A project designed to examine, for the first time, the health records of adult prisoners in Northern Ireland and their linkage to other available health data: the test case of prisoner post-release mortality risk

## Objectives

The linkage of routinely collected administrative data for research purposes has the potential to improve knowledge and public benefit. We describe a novel data linkage study between the Northern Ireland (NI) Healthcare in Prisons and Business Services Organisation (BSO). This work is undertaken within the Administrative Data Research Centre-NI (ADRC-NI).

## Approach

This joint project between ADRC-NI Queen’s University Belfast and NI Healthcare in Prisons (South Eastern Health and Social Care Trust) will test linkage of prisoner health records to health data held in the BSO and the potential to generate a population-based cohort for a retrospective analysis of prisoner health (2012-2021) that will attempt to characterise prisoners according to socio-demographic, health and committal factors, compare post-release mortality rates with a reference group from the NI population using indirect standardisation and estimate post-release mortality risk using Cox proportional hazards models.

## Results

Using novel data-linkages, a dataset will be created to examine the health of prisoners (and former prisoners) in NI. Ethics and governance approvals are in place for this data-linkage. The linkage will be undertaken via the Honest Broker Service (HBS) in NI and the dataset will be accessed in the safe setting at the BSO. The processes involved, experiences including significant delays or difficulties, and recommendations for future data-linkage studies will be discussed. In addition, a key deliverable of this project will be an assessment of access and linkage capabilities of the prisoner health data, with metadata created and made available to future researchers. In addition, we plan to present preliminary results relating to the test research question.

## Conclusion

We will describe the processes involved and first-hand research experience in the development of a novel data-linkage project, in addition we will detail access and linkage capabilities in relation to this new dataset to examine health in prisoners (and former prisoners) in NI.

